# Gastric Emphysema a Spectrum of Pneumatosis Intestinalis: A Case Report and Literature Review

**DOI:** 10.1155/2014/891360

**Published:** 2014-07-01

**Authors:** Guillermo López-Medina, Roxana Castillo Díaz de León, Alberto Carlos Heredia-Salazar, Daniel Ramón Hernández-Salcedo

**Affiliations:** ^1^Hospital Angeles Clinica Londres, Durango No. 50, Roma Norte, Cuauhtémoc, 06700 Ciudad de México, DF, Mexico; ^2^Hospital Angeles Mocel, Gregorio V. Gelati 29, San Miguel Chapultepec, Miguel Hidalgo, 11850 Ciudad de México, DF, Mexico

## Abstract

The finding of gas within the gastric wall is not a disease by itself, rather than a sign of an underlying condition which could be systemic or gastric. We present the case of a woman identified with gastric emphysema secondary to the administration of high doses of steroids, with the purpose of differentiating emphysematous gastritis versus gastric emphysema due to the divergent prognostic implications. Gastric emphysema entails a more benign course, opposed to emphysematous gastritis which often presents as an acute abdomen and carries a worse prognosis. Owing to the lack of established diagnostic criteria, computed tomography is the assessment method of choice. Currently no guidelines are available for the management of this entity, since the evidence is limited to a few case series and a considerable number of single case reports.

## 1. Introduction

Radiologic detection of gas within the wall of the gastric chamber is not an entity per se but a sign of an underlying disease [1]. Described for the first time in 1889 by Fraenkel, a variety of names have been given to this condition: interstitial emphysema, intramural emphysema, gastric emphysema, emphysematous gastritis, gastric pneumatosis, and pneumatosis cystoides intestinalis; however, based on underlying cause and anatomical situation they are considered distinct pathologies carrying for a different treatment and prognosis [1, 2].

## 2. Case Report

**Figure 1:**
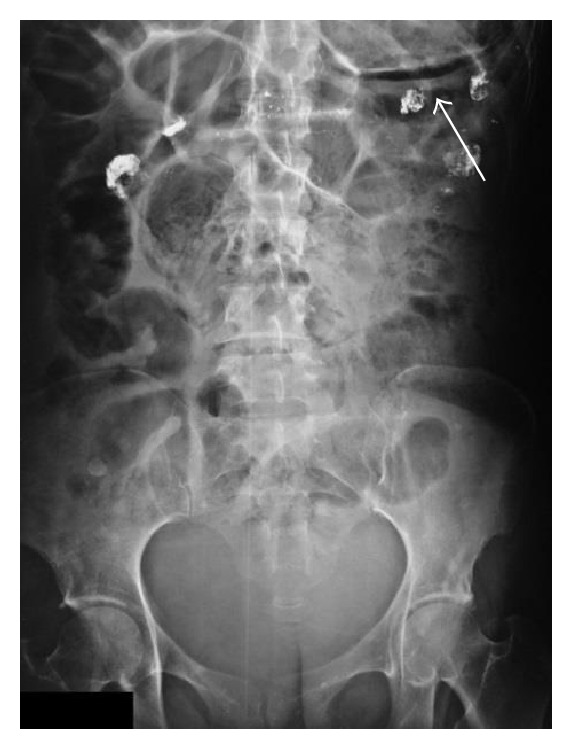
Plain abdominal X-ray. Radiolucent image in the upper left abdominal quadrant, showing the presence of air within the wall of the stomach (arrow).

**Figure 2:**
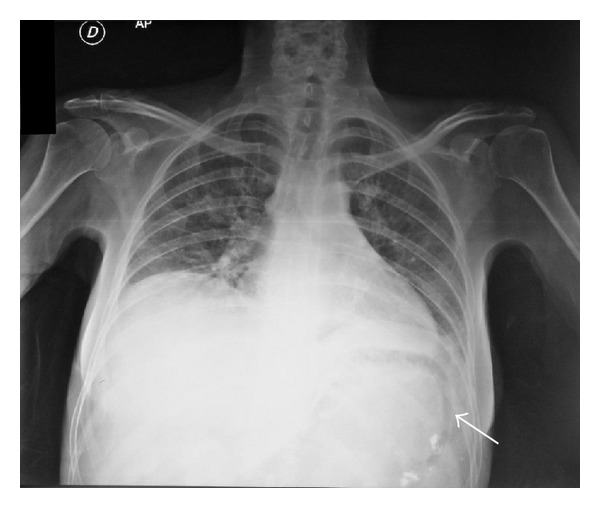
Plain chest X ray. Radiolucent image below the left diaphragm showing the presence of air within the wall of the stomach (arrow).

**Figure 3:**
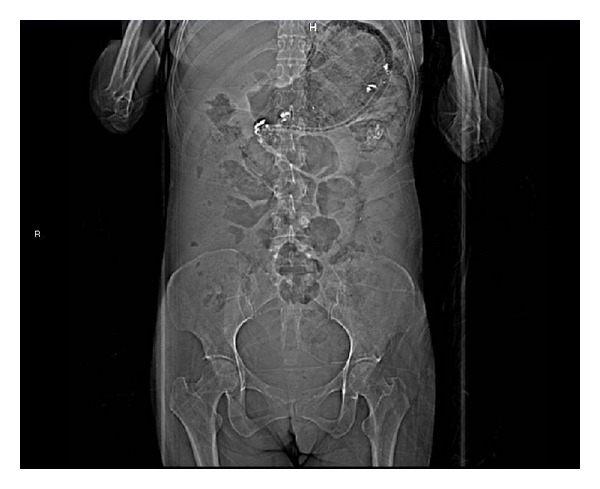
Abdominal scout image from a computed tomography.

**Figure 4:**
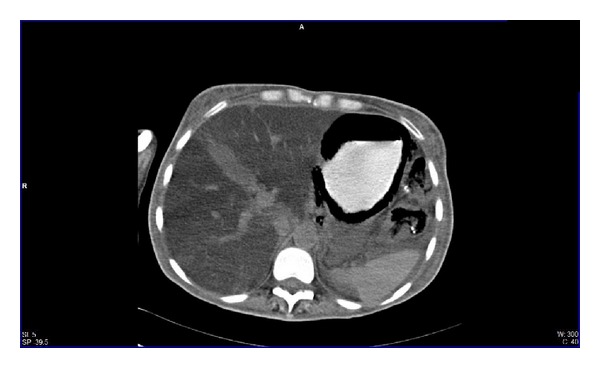
Computed axial tomography of the abdomen with oral contrast agent enhancing the stomach.

**Figure 5:**
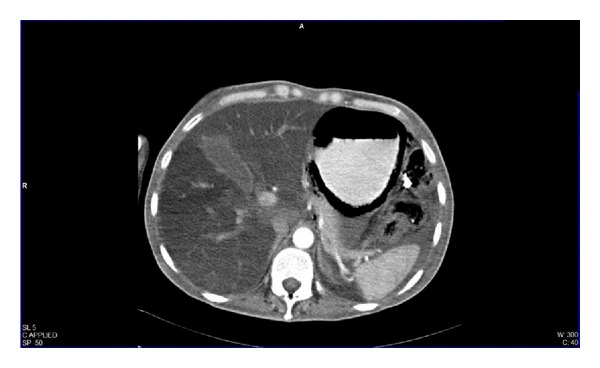
Computed axial tomography of the abdomen with oral and intravenous contrast agent enhancing the stomach during the arterial phase.

We present a 44-year-old woman with history of mixed connective tissue disease, who was hospitalized due to exacerbation of her underlying condition for which she was treated with high dose pulses of methylprednisolone. Two days after discharge she arrives back to the emergency room, complaining mainly of abdominal pain located on upper quadrants, not related to food ingestion, associated with vomiting of gastric content in four occasions and one liquid stool free from mucus or blood. At admission with stable vital signs, without acute abdomen or other relevant findings revealed on physical examination, stool specimens, blood analyses, and cultures analyses were requested in search for infectious origin, resulting negative. The plain abdominal X-ray showed a radiolucent image in the left upper quadrant (Figure 1), which was also observed on the plain chest X-ray (Figure 2), subsequently a computed tomography (CT) scan with double contrast of the abdomen, documented the presence of air in the gastric wall (Figures 3, 4, and 5).

Fasting was indicated as well as management with proton bomb inhibitor and broad spectrum antibiotic ampicillin/sulbactam. After twelve hours of close monitoring, the patient persisted nauseous and vomiting, therefore, underwent a liberating mucotomy via superior endoscopy, without complications and minimum bleeding. However, 24 hours later the patient continued presenting abdominal pain and under the suspicion of gastric perforation, due to the presence of right subdiaphragmatic air on a chest X-ray, she was taken to the operating room for an exploratory laparotomy revealing free hematic fluid in the abdominal cavity; methylene blue dye was instilled into the gastric lumen without evidence of dye extravasation, concluding the absence of perforation, considering the procedure complete. Afterwards the evolution was torpid showing signs of rheumatologic activity associated to acute pulmonary deterioration that led to the patient's death due to a possible hemorrhagic alveolitis.

## 3. Discussion

The pathogenesis of this disease has been debated for decades; however, it may be approached through the questions, where did the gas come from? And how did it get there? Based on these are how three mechanisms of origin are proposed: (1) intraluminal, (2) bacterial production, and (3) gas of pulmonary origin [3].

Intestinal pneumatosis is referred to the presence of gas within the wall of the gastrointestinal tract and it can appear in any site from stomach to the rectum [4]. The stomach is the least common site of presentation; one retrospective study identified only 18 cases during a period of 15 years [5]. Another study in 2004 by Hawn MT, Canon CL, and Lockhart ME et al. reported a casuistry of 86 patients with intestinal pneumatosis, being the colonic localization the most frequent counting for 50%, while the gastric location counted hardly 9%, with a mortality rate of 38% reported for this last one [6]. Emphasizing on the gastric chamber, it can be classified into two categories: emphysematous gastritis and gastric emphysema or gastric pneumatosis [7]. Nevertheless there are no clear and universal definitions available to distinguish each.

It has been postulated that emphysematous gastritis is produced by gas forming bacteria and gastric emphysema by air dissecting the wall [8], through several mechanisms either traumatic, mechanic, or inflammatory [9]; it is important to mention that emphysematous gastritis and gastric emphysema entail contrasting prognosis, the latter being more benign [8].

When the origin of the gas is intraluminal, pneumatosis can occur even alongside an intact mucosa, with intraluminal high pressure accounted as the responsible mechanism. In the context of an injured mucosa, because of trauma or inflammation, a normal intraluminal pressure may be present or a combination of both [3].

In the case gas derives from bacteria, the theory of counterperfusion-supersaturation has been postulated, and it sustains that the production of nitrogen by intraluminal bacteria overflows the plasma concentration producing a plasma-intraluminal gradient and causing a diffusion of nitrogen into submucosal vessels which would explain the gas pattern found through the blood vessels in the border of the mesentery [3].

A pulmonary source has also been debated. The proposed theory is that air travels from an alveolar rupture into the blood vessels up to the gastrointestinal tract. Nevertheless the absence of interstitial emphysema in the mesentery has called this theory into question, giving place to a hypothesis of an increase in intraabdominal pressure hence intraluminal pressure, frequently seen in chronic cough patients causing transmural migration of air [3].

Resuming the etiopathogenesis of this entity, some causes can be mentioned in referral to gastric processes associated to injury of the mucous wall of the stomach. It is well known that acute gastric distension can result in gastric emphysema, emphysematous gastritis, or necrosis. Massive distension causes ischemia with extension of intraluminal gas in to the wall [10]. The ingestion of caustic substances, primarily acids more than alkalis promote corrosive lesions altering the gastric wall in various degrees of depth [11]. Also there have been reported cases of caustic gastritis coursing without gastric emphysema and yet gas within portal venous system [12]. Perforating ulcers, upper endoscopy procedures, such as argon plasma coagulation, installation of intragastric catheters, or biliary stents, are diverse precedents of lesion that can complicate with mobilization of intraluminal gas to submucous spaces [11]. Evidence exists of gastric bezoars associated with this disease as well as distension due to the administration of positive pressure through a bag-mask device during cardiopulmonary resuscitation [13–15], in addition to a case secondary to a diabetic gastropathy in a patient with type 1 diabetes [16].

Pneumatosis can also result from a variety of extragastric processes, which favor air migration thru the wall of the colon despite maintaining intact. That being said we can include chronic pulmonary obstructive disease, polymyositis, perforated appendicitis, small bowel volvulus, intestinal infarction, gangrenous cholecystitis, superior mesenteric artery syndrome, cholangiocarcinoma, parastomal hernia, and multiple episodes of vomiting. In relation to drug related background, it has been linked to chemotherapy agents such as cyclophosphamide, adriamycin, and vincristine, in addition to high doses of dexamethasone in one case report [5, 17–23].

Clinical manifestations are usually nonspecific, presenting with nausea, vomit occasionally resistant to antiemetics, mild to severe abdominal pain, abdominal distension, haematemesis, or melena; presentation as an acute abdomen is rare [2]. Most frequently reported cases follow an acute- subacute course [5, 16, 17, 23] and findings at physical examination rarely support the diagnosis [3].

Among the image battery available, computed tomography (CT) of the abdomen is the method of choice since it can detect a minimum amount of air inside the wall of the gastrointestinal tract and evaluate the abdominal cavity. Gastric emphysema is presented as a hypodense lineal or curve fringe on the gastric wall along with distension, without evidence of thickening of the wall. Occasionally pneumoperitoneum may be detected, in contrast to emphysematous gastritis where a streaky and linear pattern distribution of air and gastric wall thickening are characteristic, or air in some other bowel or biliary tract can be found [5, 17, 24]. In a simple X-ray of the abdomen, stomach is distended and outlined with linear gas shadows in the gastric wall. These stripes or streaks can be single or double, with round areas of radiolucency a few millimeters wide and parallel to the border of the stomach [17]. Within endoscopic findings we can come across a pebble-like gastric mucous that only traduces the presence of air bubbles [25].

There is no available standard treatment for this condition; most of the reported cases have been treated in a conservative manner. A retrospective study by Morris et al. [26] included 97 patients with intestinal pneumatosis, 46% involving colon, 27% small bowel, 14% affecting the whole gastrointestinal tract including the portal venous system, 7% small bowel and colon, and 5% located on the stomach; they reported a mortality of 16% in the group who received surgical treatment contrasting with a 6% mortality rate reported for the group who received conservative treatment. In stable patients a clinical improvement or resolution of the emphysema within 72 hours has been reported with the use of nasogastric catheter for decompression [10]. Algorithms for the management of intestinal pneumatosis have been proposed, however, without emphasis on gastric emphysema so their application is limited [27, 28].

The prognosis of gastric emphysema is usually benign with spontaneous resolution even without a specific treatment [3]. More so, a reported case of recurrent gastric emphysema by two distinct mechanisms, where managed in a conservative way obtaining a favorable outcome [29]. Nevertheless mortality associated to emphysematous gastritis is documented from 61% as far as 100% [2, 9].

## 4. Conclusion

Gastric emphysema and emphysematous gastritis are spectrum of intestinal pneumatosis; both belong to the least common forms of presentation, with different etiopathogenic possibilities (traumatic, inflammatory, or mechanic), systemic impact, intraabdominal findings, and prognosis and with a distinctive feature of gastric emphysema that in most cases yields more indolent behavior. It is maintained as a misunderstood pathology, in great part since most of the evidence derives from single case reports and series in a significant number of clinical scenarios.

The main suspicion in the presented case was an inflammatory cause which was sustained by the presence of hematic ascites, not rare in patients with connective tissue diseases. The implemented measures of endoscopic mucotomy plus coverage by board spectrum antibiotics turned out in a positive impact regarding the gastrointestinal aspect, unlike the rest of the progression of her base rheumatologic disease.

## References

[1] Agha FP (1984). Gastric emphysema: an etiologic classification. *Australasian Radiology*.

[2] Loi TH, See J, Diddapur RK, Issac JR (2007). Emphysematous gastritis: a case report and a review of literature. *Annals of the Academy of Medicine Singapore*.

[3] St Peter SD, Abbas MA, Kelly KA (2003). The spectrum of pneumatosis intestinalis. *Archives of Surgery*.

[4] Donovan S, Cernigliaro J, Dawson N (2011). Pneumatosis intestinalis: a case report and approach to management. *Case Reports in Medicine*.

[5] Majumder S, Trikudanathan G, Moezardalan K, Cappa J (2012). Vomiting-induced gastric emphysema: a rare self-limiting condition. *The American Journal of the Medical Sciences*.

[6] Hawn MT, Canon CL, Lockhart ME (2004). Serum lactic acid determines the outcomes of CT diagnosis of pneumatosis of the gastrointestinal tract. *The American Surgeon*.

[7] D'Cruz R, Emil S (2008). Gastroduodenal emphysema. *Journal of Pediatric Surgery*.

[8] Pauli EM, Tomasko JM, Jain V, Dye CE, Haluck RS (2011). Multiply recurrent episodes of gastric emphysema. *Case Reports in Surgery*.

[9] Krier M, Jeffrey RB, Banerjee S (2009). Troubles with stomach bubbles? or not?. *Digestive Diseases and Sciences*.

[10] Khan SA, Boko E, Khookhar HA, Woods S, Nasr AH (2012). Acute gastric dilatation resulting in gastric emphysema following postpartum hemorrhage. *Case Reports in Surgery*.

[11] Johnson PT, Horton KM, Edil BH, Fishman EK, Scott WW (2011). Gastric pneumatosis: the role of CT in diagnosis and patient management. *Emergency Radiology*.

[12] Lewin M, Pocard M, Caplin S, Blain A, Tubiana J-M, Parc R (2002). Benign hepatic portal venous gas following caustic ingestion. *European Radiology*.

[13] Chintapalli KN (1994). Gastric bezoar causing intramural pneumatosis. *Journal of Clinical Gastroenterology*.

[14] Reuter H, Bangard C, Gerhardt F, Rosenkranz S, Erdmann E (2011). Extensive hepatic portal venous gas and gastric emphysema after successful resuscitation. *Resuscitation*.

[15] Lai C, Chang W, Liang P, Lien W, Wang H, Chen W (2005). Pneumatosis intestinalis and hepatic portal venous gas after CPR. *The American Journal of Emergency Medicine*.

[16] Cherian SV, Das S, Khara L, Garcha AS (2011). Gastric emphysema associated with diabetic gastroparesis. *Internal Medicine*.

[17] Zenooz NA, Robbin MR, Perez V (2007). Gastric pneumatosis following nasogastric tube placement: a case report with literature review. *Emergency Radiology*.

[18] Sakamoto Y, Mashiko K, Matsumoto H, Hara Y, Kutsukata N, Yamamoto Y (2006). Gastric pneumatosis and portal venous gas in superior mesenteric artery syndrome. *Indian Journal of Gastroenterology*.

[19] Tuck JS, Boobis LH (1987). Interstitial emphysema of the stomach due to perforated appendicitis. *Clinical Radiology*.

[20] Lim JE, Duke GL, Eachempati SR (2003). Superior mesenteric artery syndrome presenting with acute massive gastric dilatation, gastric wall pneumatosis, and portal venous gas. *Surgery*.

[21] Zander T, Briner V, Buck F, Winterhalder R (2007). Gastric pneumatosis following polychemotherapy. *European Journal of Internal Medicine*.

[22] Ilyas C, Young AL, Lewis M, Suppia A, Gerotfeke R, Perry EP (2012). Parastomal hernia causing gastric emphysema. *Annals of the Royal College of Surgeons of England*.

[23] Chou J, Tseng Y, Tseng C (2010). A rare complication of chemotherapy in a 56-year-old patient. *Gastroenterology*.

[24] Chang Y-L, Lu Y-Y, Huang C-C, Chiu H-H (2011). Gastrointestinal: gastric emphysema and pneumoperitoneum with spontaneous resolution. *Journal of Gastroenterology and Hepatology*.

[25] Grayson DE, Abbott RM, Levy AD, Sherman PM (2002). Emphysematous infections of the abdomen and pelvis: a pictorial review. *Radiographics*.

[26] Morris MS, Gee AC, Cho SD (2008). Management and outcome of pneumatosis intestinalis. *The American Journal of Surgery*.

[27] Greenstein AJ, Nguyen SQ, Berlin A (2007). Pneumatosis intestinalis in adults: Management, surgical indications, and risk factors for mortality. *Journal of Gastrointestinal Surgery*.

[28] Wayne E, Ough M, Wu A (2010). Management algorithm for pneumatosis intestinalis and portal venous gas: treatment and outcome of 88 consecutive cases. *Journal of Gastrointestinal Surgery*.

[29] Kalina M, Rubino M (2009). Recurrent gastric emphysema. *American Surgeon*.

